# Acquired resistance to jadomycin B in human triple-negative breast cancer cells is associated with increased cyclooxygenase-2 expression

**DOI:** 10.1016/j.jpet.2025.103565

**Published:** 2025-03-27

**Authors:** Brendan T. McKeown, Brandon Groves, David L. Jakeman, Kerry B. Goralski

**Affiliations:** 1 Department of Pharmacology, Faculty of Medicine, Dalhousie University, Halifax, Nova Scotia, Canada; 2 Beatrice Hunter Cancer Research Institute, Halifax, Nova Scotia, Canada; 3 Department of Chemistry, Faculty of Sciences, Dalhousie University, Halifax, Nova Scotia, Canada; 4 College of Pharmacy, Faculty of Health, Dalhousie University, Halifax, Nova Scotia, Canada; 5 Division of Pediatric Hematology and Oncology, Department of Pediatrics, IWK Health Centre, Halifax, Nova Scotia, Canada

**Keywords:** Breast cancer, Multidrug resistance, Cyclooxygenase, Topoisomerase, Jadomycin, ATP-binding cassette transport

## Abstract

Jadomycin B, produced by the soil bacterium *Streptomyces venezuelae* ISP5230*,* induces cytotoxicity in human breast cancer cells in vitro and has antitumoral effects in animal models. In models of multidrug-resistant, triple-negative breast cancer, jadomycin B has shown promise as it is not a substrate of ABCB1 and ABCG2 drug efflux transporters. The generation of reactive oxygen species and inhibition of topoisomerases are potential mechanisms of jadomycin B–mediated DNA damage and apoptosis. However, the mechanisms of jadomycin B’s anticancer activity have not been fully elucidated. By gradually exposing MDA-MB-231 triple-negative human breast cancer cells to jadomycin B, we hypothesized that resistance could be selected to further understand jadomycin B’s pharmacological mechanisms. A 3-fold increase in the jadomycin B IC_50_ was observed in MDA-MB-231 cells exposed to increasing jadomycin B concentrations (0–3 *μ*M) over 7 months, herein 231-JB cells. The 231-JB cells were cross-resistant to jadomycin F and S but not to the comparator drugs mitoxantrone, doxorubicin, and SN-38. The 231-JB cells did not have increased mRNA expression of *topoisomerase-2* nor *ABCB1* and *ABCG2*. Cyclooxygenase-2 (COX-2) increased by 25-fold, but expression of prostaglandin E_2_ receptor 4 did not significantly change. Cotreatment with celecoxib (15–45 *μ*M), a COX-2 inhibitor, resensitized the 231-JB cells to jadomycin B (IC_50_ = 1.41 ± 0.24 to 0.75 ± 0.31 *μ*M vs 2.28 ± 0.54 with 0 *μ*M celecoxib). To our knowledge, this work represents the first report of the involvement of COX-2 in jadomycin B activity in vitro, proving to be an exciting new target for the exploration of jadomycin B anticancer activity.

**Significance Statement:**

Cyclooxygenase-2 (COX-2), the rate-limiting enzyme in prostaglandin production, is associated with procancer signaling. COX-2, ABCB1, and ABCG2 overexpression are typically correlated in cancer, contributing to chemotherapy resistance. We observed increased COX-2, but not ABCG2 or ABCB1, expression in 231-JB cells. This indicates that jadomycin B triggers a distinct resistance mechanism. The COX-2 inhibitor celecoxib reversed jadomycin B resistance in 231-JB cells. As such, 231-JB cells represent an important model for COX-2 signaling in breast cancer and jadomycin B mechanism of action.

## Graphical abstract

**Figure:**
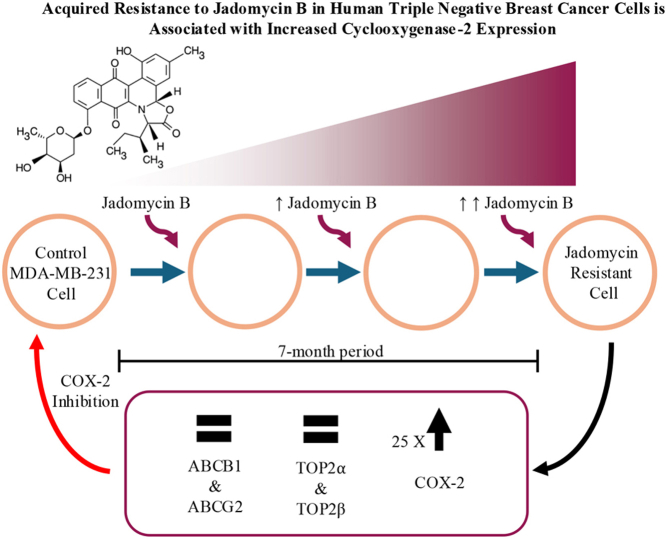

## 1 Introduction

Breast cancer is the most commonly diagnosed cancer in Canadian and American women (Brenner et al, 2022; Siegel et al, 2023), recently surpassing lung cancer as the most common cancer worldwide (Sung et al, 2021). Approximately 20%–30% of women diagnosed with breast cancer are expected to progress to metastatic disease, reducing the median survival to 2–3 years (Morris et al, 2009; Rivera and Gomez, 2010). Triple-negative breast cancers make up 10%–20% of advanced breast cancers, resulting in loss of efficacy of treatments targeting estrogen or progesterone receptors and human epidermal growth factor receptor 2 (Kumar and Aggarwal, 2016; Cardoso et al, 2017). Treatment of triple-negative breast cancer instead relies on cytotoxic chemotherapies (Cardoso et al, 2017).

Further complicating treatment, chronic exposure to cytotoxic agents increases risk for development of multidrug resistance, which leads to difficulties in treatment and curability (Morris et al, 2009; Rivera and Gomez, 2010). One of the most well-documented mechanisms driving multidrug resistance involves the increased function of ATP-binding cassette (ABC) drug efflux transporters such as P-glycoprotein (ABCB1), multidrug resistance–associated protein (ABCC1), and breast cancer resistance protein (ABCG2) (Szakács et al, 2006; Morris et al, 2009; Robey et al, 2018). Unfortunately, inhibition of ABC transporters has not shown improved outcomes or has led to unacceptable adverse effects (Pusztai et al, 2005; Amiri-Kordestani et al, 2012; Callaghan et al, 2014). New drugs that can avoid drug efflux while acting on validated anticancer pathways are therefore needed to fulfill an unmet need for patients with metastatic, triple-negative breast cancer.

**Fig. 1:**
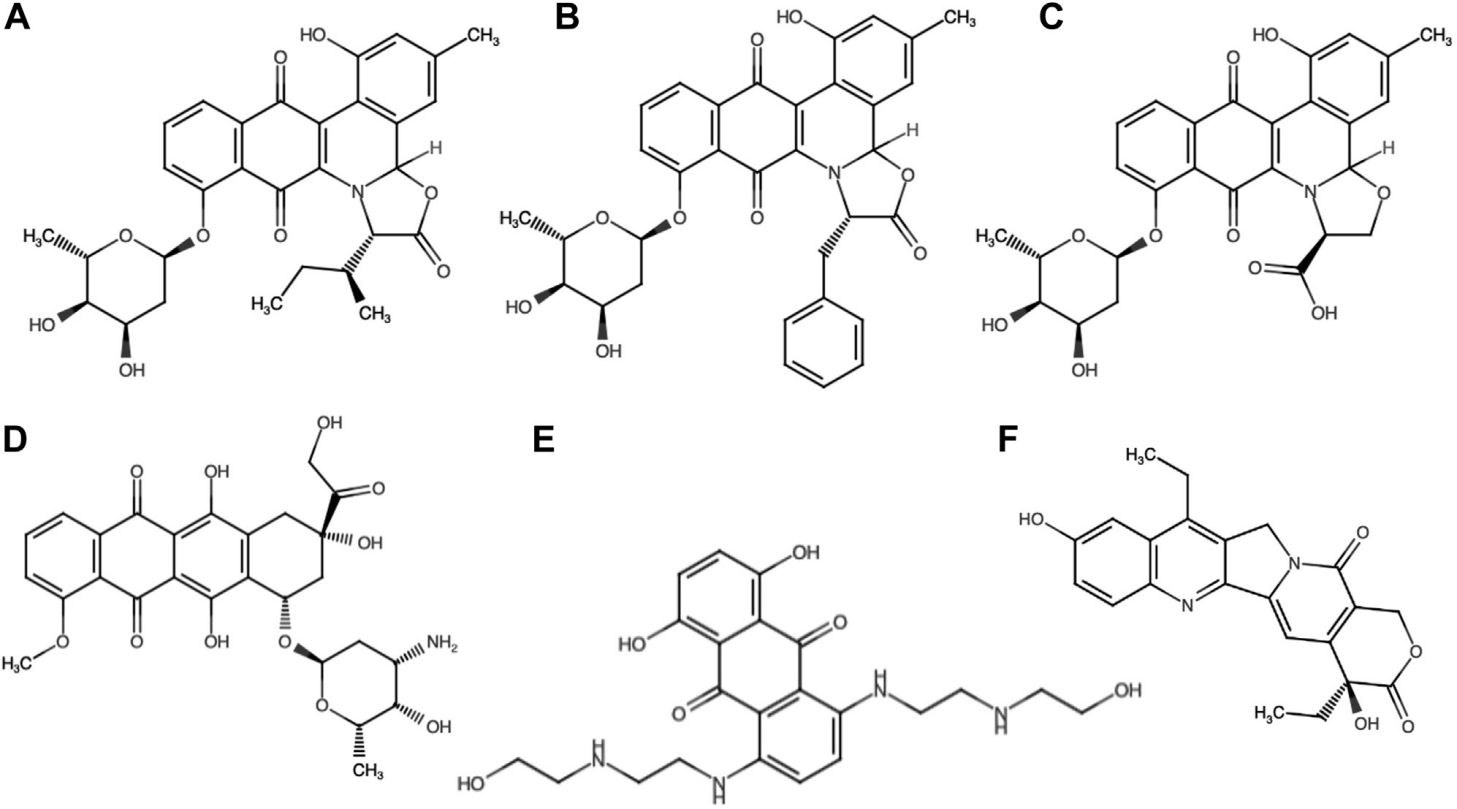
Chemical structures of jadomycins and comparator topoisomerase targeting agents: (A) jadomycin B, (B) jadomycin F, (C) jadomycin S, (D) doxorubicin, (E) mitoxantrone, and (F) SN-38 are used in this work.

Natural products from microbial, plant, and marine sources have been important sources of novel therapeutics for cancer (Demain and Vaishnav, 2011). Jadomycins (Fig. 1, A–C) are secondary metabolites produced under stress conditions by the bacterium *Streptomyces venezuelae* ISP5230 (Jakeman et al, 2006). By adjusting the amino acid content of the growth medium, 70 different jadomycins have been produced with a range of anticancer activities (MacLeod et al, 2018; Bonitto et al, 2021). Jadomycins have previously been shown to have equivalent potency in breast cancer cells following the development of resistance to ABCB1 and ABCG2 transporter substrates (Issa et al, 2014; Hall et al, 2015, 2017). Additionally, jadomycins are equally potent against estrogen receptor–positive, progesterone receptor–positive, and human epidermal growth factor–overexpressing breast cancer cells (Hall et al, 2015; Bonitto et al, 2021). Recently, a pilot study on the pharmacokinetics of jadomycin B in animals has demonstrated a good safety profile at concentrations sufficient to decrease primary tumor volume (McKeown et al, 2022). These factors make jadomycins an attractive group of compounds to research for the treatment of advanced breast cancers.

The mechanism by which jadomycins exert their anticancer effect remains to be fully elucidated. Previously, jadomycins have been demonstrated to elicit apoptotic cell death through the accumulation of DNA damage in breast cancer cells (Fu et al, 2008; Hall et al, 2017). There have been several proposed mechanisms through which this is initiated, including the generation of reactive oxygen species, aurora B kinase inhibition, and poisoning of topoisomerase-2 (TOP2) (Fu et al, 2008; Issa et al, 2014; Hall et al, 2015, 2017; Bonitto et al, 2021; McKeown and Goralski, 2025). The TOP2 and aurora B kinase effects, however, occur at concentrations greater than the IC_50_ values commonly reported for jadomycins’ anticancer effect (Bonitto et al, 2021). While generation of reactive oxygen species does occur at lower concentrations, the inhibition of reactive oxygen species production by *N*-acetylcysteine does not prevent jadomycin-induced DNA damage leading to the conclusion that reactive oxygen species are not the sole mechanism of DNA damage and apoptosis (Hall et al, 2015). The results described in our previous publication also demonstrate that jadomycin B does not completely emulate the activity of doxorubicin or mitoxantrone as a TOP2 poison (McKeown and Goralski, 2025). These differences suggest a different primary mechanism of action may be responsible for the cytotoxic effect of jadomycin B.

There has been specific interest in the development of jadomycin-resistant models of breast cancer for the purpose of elucidating mechanism of action (Bonitto et al, 2021). Herein, we hypothesized that the development of a jadomycin-resistant triple-negative breast cancer cell line could be used to identify genes of interest involved in the mechanism by which jadomycin B exerts its effect. Given the previous interest in the TOP2 poisoning effect, it was also important to include topoisomerase targeting agents, which are also known drug efflux transporter substrates as comparator drugs (Fig. 1, D–F) in the present study (Bonitto et al, 2021).

## 2 Materials and methods

### 2.1 Chemical and biological materials

Dulbecco’s modified Eagle’s medium, molecular-grade water, FBS, penicillin and streptomycin, sodium pyruvate, trypsin, PBS, superscript II reverse transcriptase, random hexamer primer, RNase OUT, dithiothreitol, deoxynucleoside triphosphates, and reverse transcriptase buffer were purchased from Thermo Fisher Scientific. Jadomycin B, mitoxantrone, doxorubicin, celecoxib, naproxen, thiazolyl blue methyl tetrazolium bromide (MTT), SN-38, bovine serum albumin, sodium hydroxide, copper(II) sulfate pentahydrate, potassium sodium tartrate tetrahydrate, Folin and Ciocalteu phenol reagent, Tris-HCl, SDS, bromophenol blue, glycerol, *β*-mercaptoethanol, acrylamide, *N*ʹ,*N*ʹ-methylenebisacrylamide, ammonium persulfate, *N*,*N*,*N*ʹ,*N*ʹ-tetramethylethylenediamine, glycine, NaCl, KCl, Tween-20, methanol, agarose, ethidium bromide, DMSO, apramycin sulfate, MgSO_4_, (NH_4_)_6_Mo_7_O_24_, K_2_HPO_4_, KH_2_PO_4_, sulfuric acid, and dichloromethane were purchased from Sigma Aldrich. *D*-maltose, yeast extract, malt extract, agar, 3-(*N*-morpholino)propanesulfonic acid, *L*-serine, and *L*-phenylalanine were purchased from BioShop Canada. FeSO_4_, CaCL_2_, ZNSO_4_, MnSO_4_, H_3_BO_3_, and glucose were purchased from Fisher Scientific Canada. Anhydrous ethanol was purchased from Commercial Alcohols. Nitrocellulose membranes were purchased from GE Healthcare. Immunoblot blocking solution, protein ladder, and all secondary antibodies were purchased from LI-COR Biosciences. Primary antibodies were purchased from Abcam, Inc.

### 2.2 Production of jadomycins

Jadomycin F (*L*-phenylalanine), and jadomycin S (*L*-serine) were isolated and characterized as previously described (Jakeman et al, 2006, 2009; Dupuis et al, 2011; Issa et al, 2014; Robertson et al, 2018). These jadomycin analogs were chosen, along with jadomycin B (*L*-isoleucine), as they represent 3 categories spanning the diversity of jadomycins: jadomycins with hydrophobic aliphatic side chains (B, isoleucine), hydrophobic aromatic side chains (F, phenylalanine), and hydrophilic side chains (S, serine).

### 2.3 Cell lines: development and selection of resistance

All human breast cancer cell lines were cultured in 75-mm^2^ tissue-culture flasks (Corning, Inc) in phenol red-free Dulbecco’s modified Eagle’s medium supplemented with 10% FBS, 100 IU/mL penicillin and 250 *μ*g/mL streptomycin, and 1 mM sodium pyruvate (complete medium) at 37 °C in a humidified atmosphere of 5% CO_2_ (standard conditions) as previously described (Hall et al, 2017). All cells tested negative for mycoplasma contamination using a MycoAlert mycoplasma detection kit (Lonza Biosciences).

Control MDA-MB-231 human triple-negative breast cancer cells (231-CON) cells were kindly provided by Drs David Hoskin and Anna Greenshields (Dalhousie University, Halifax, Nova Scotia, Canada). Polyclonal jadomycin-resistant MDA-MB-231 cells (231-JB) and mitoxantrone-resistant MDA-MB-231 cells (231-MITX) were generated in-house using previously described methods (Schneider et al, 1994; Hall et al, 2017). Michigan Cancer Foundation-7 (MCF-7) hormone receptor positive human breast cancer cells were kindly provided by Dr Aik Jiang Lau (Dalhousie University). Briefly, increased resistance to jadomycin B or mitoxantrone was selected for by gradually increasing concentrations of jadomycin B or mitoxantrone in complete medium over 7 months until a final concentration of 3.0 *μ*M jadomycin B or 0.015 *μ*M mitoxantrone, respectively, was reached. Cells were not exposed to mutagens prior to selection nor clonally isolated after selection. Following selection, 231-JB and 231-MITX cells were passaged in drug-free complete medium and remained stably resistant to jadomycin B or mitoxantrone, respectively. To prevent reversion of resistance, 231-JB and 231-MITX cells were maintained in complete medium containing 3.0 *μ*M jadomycin B or 0.015 *μ*M mitoxantrone, respectively. Resistant cells were cultured in drug-free complete medium for 1 week prior to experiments.

Mitoxantrone-resistant cells were generated as a comparator because they were expected to be ABCG2 overexpressing as previously observed in other mitoxantrone-resistant cell lines and serve a control for ABCG2 drug efflux transport (Litman et al, 2000; Issa et al, 2014). Mitoxantrone is also a relevant control based on the potential TOP2 inhibitory activity of jadomycins (Martinez-Farina et al, 2015; Hall et al, 2017).

### 2.4 MTT viability assays

MTT assays were used to evaluate relative anticancer activity of jadomycins B, S, and F (0.2–25 *μ*M) and the ABC transporter substrates mitoxantrone (0.004–50 *μ*M), doxorubicin (0.08–15 *μ*M), and SN-38 (0.08–20 *μ*M) in 231-CON, 231-JB, and 231-MITX cells for 48 hours as previously described (Issa et al, 2014). Each data point consisted of an average value from quadruplicate technical replicates and each experiment was repeated in quadruplicate.

### 2.5 RNA isolation, reverse transcription, and quantitative real-time polymerase chain reaction

Total RNA was isolated from lysates of 231-CON, 231-JB, and 231-MITX cells using the Aurum Total RNA Mini Kit (Bio-Rad Laboratories) as per manufacturer instructions. Cells were seeded into 6-well plates (Corning, Inc) at 200,000 cells/well in 2 mL of drug-free complete medium and allowed to adhere under standard conditions. Cells were then either allowed to grow for 48 hours and collected to generate control samples or exposed in triplicate for 48 hours with either DMSO vehicle control or jadomycin B (2.5–5.0 *μ*M). Triplicate well lysates were pooled to generate a single sample. RNA samples were quantified using a BioTek Synergy HT plate reader using Gen5 v2.01 software (Agilent Technologies) in a UV transparent microplate (Corning, Inc) with path length correction. Isolated RNA (0.5 *μ*g) was reverse transcribed to complementary DNA using Super Script II Reverse Transcriptase and a TProfessional Basic 96 Thermocycler (Montreal Biotech, Inc). Complementary DNA was amplified by quantitative polymerase chain reaction (PCR) using 125-nM gene-specific primers (Supplemental Table 1) in a total volume of 20 *μ*L using Sso Advanced Universal SYBR Green Supermix (Bio-Rad Laboratories) and a StepOne Plus real-time PCR thermocycler using StepOne Software v2.1 (Applied Biosystems) in duplicate for each primer set and averaged. Each experiment was repeated in triplicate. Gene expression was normalized using the average of 3 housekeeping genes (glyceraldehyde-3-phosphate dehydrogenase, *β*-actin, and peptidylprolyl isomerase A [alternatively known as cyclophilin A]) via the ΔΔC_t_ method (Livak and Schmittgen, 2001).

### 2.6 RT^2^ profiler PCR array

Total RNA was collected and converted to cDNA as above from 231-CON and 231-JB cells, which were not exposed to any small molecule intervention. Human Cancer Drug Targets RT^2^ Profiler PCR Arrays (Qiagen) were conducted as per manufacturer instructions using a StepOne Plus real-time PCR thermocycler using StepOne Software v2.1 (Applied Biosystems).

### 2.7 Immunoblot analysis

231-CON and 231-JB cells were seeded into 6-well plates (Corning, Inc) at 200,000 cells per well and left to adhere overnight in complete medium at standard conditions. Cells were then either collected to generate control samples or exposed in duplicate for 48 hours with either drug-free complete medium, DMSO vehicle control, or jadomycin B (2.5–5.0 *μ*M). Duplicate wells were pooled to generate a single sample and lysed using RIPA Buffer (Santa Cruz Biotechnologies, Inc) as per manufacturer instructions. Protein content in the lysate was measured using the Lowry Assay (Lowry et al, 1951). Immunoblotting was conducted as previously described (McKeown et al, 2014). Briefly, 25 *μ*g protein samples were prepared in a standard Laemmli buffer consisting of 50 mM Tris-HCl (pH 6.8), 2% SDS, 0.1% bromophenol blue, 10% glycerol, and 100 mM *β*-mercaptoethanol and then boiled for 3 minutes (Laemmli, 1970). Electrophoresis through 12.5% SDS-PAGE gels was conducted in duplicate for each sample, and these were transferred to nitrocellulose membranes. Membranes were incubated in LI-COR blocking solution (Mandel Scientific) overnight at 4 °C and then incubated in a 1:500 or 1:1000 dilution of primary antibody (Supplemental Table 2) for 1 hour at room temperature, followed by washing 3 times with TBS-Tween (0.1% v/v) for a total of 30 minutes and incubation in a 1:10,000 dilution of secondary antibody (Supplemental Table 2) for 1.25 hours at room temperature, in the dark. After incubation with secondary antibodies membranes were again washed 3 times with TBS-Tween (0.1% v/v) for a total of 30 minutes and then visualized. For visualization and quantification, membranes were scanned at 700 nm and 800 nm using a LI-COR Odyssey CLx Imager (Mandel Scientific) and analyzed using Image Studio v5.2 software (Mandel Scientific) to measure pixel intensity. Pixel intensity of each band was normalized to the intensity of the respective *β*-actin band, and these ratios were expressed as a fold change versus either drug-free 231-CON cells or vehicle control 231-CON cells. Duplicate values were averaged to generate each data point, and each experiment was replicated in quadruplicate.

### 2.8 Reversal of jadomycin B resistance with celecoxib and naproxen

Pharmacological reversal of resistance in 231-JB cells was determined by coadministration of jadomycin B (0–2.2 *μ*M) in the presence and absence of celecoxib (0–45 *μ*M), naproxen (0–1500 *μ*M), or DMSO vehicle control. Jadomycin B (0–2.2 *μ*M) was combined with DMSO vehicle control in 231-CON cells to provide a comparison with control cells. MTT viability assays were used to assess cellular viability over 48 hours in these cells in quintuplicate using the same methods described for MTT assays earlier.

### 2.9 Sensitization of 231-CON and MCF-7 cells to jadomycin B with celecoxib

231-CON cells were exposed to 0–1.5 *μ*M jadomycin B and 0–45 *μ*M celecoxib, while MCF-7 cells were exposed to 0–3 *μ*M jadomycin B and 0–30 *μ*M celecoxib. MTT viability assays were used to assess cellular viability over 48 hours in these cells in quintuplicate using the same methods described for MTT assays earlier.

### 2.10 Prostaglandin E_2_ ELISA

Levels of prostaglandin E_2_ (PGE_2_) in cell culture medium were measured using a colorimetric PGE_2_ ELISA kit (Abcam, Inc) as per manufacturer instructions. Briefly, 231-CON cells were seeded into 6-well plates (Corning, Inc) at 400,000 cells per well and left to adhere overnight in complete medium at standard conditions. Cells were exposed to jadomycin B (2.5 or 5 *μ*M) or DMSO vehicle control in complete medium for 6, 24, 48, or 72 hours, in triplicate with each measurement consisting of technical duplicates. Optical density of each sample was read at 405 nm and compared against a standard curve comprising known PGE_2_ concentrations to interpolate PGE_2_ concentrations in samples.

### 2.11 Statistical analysis

For each experiment, individual exposures were performed in triplicate, quadruplicate, or quintuplicate as described earlier. All data are expressed as mean ± SD. An unpaired *t* test was used for statistical comparisons involving 2 groups, a one-way ANOVA was used for multiple comparisons in experiments with 1 independent variable, and a two-way ANOVA was used for multiple comparisons with 2 independent variables. A Bonferroni test was used for post hoc analysis of significant ANOVA results. A difference in mean values between groups was considered significant when *P* ≤.05. All statistical tests were conducted using GraphPad Prism 9 (GraphPad).

## 3 Results

### 3.1 Assessment of resistance in 231-JB and 231-MITX cells

**Fig. 2:**
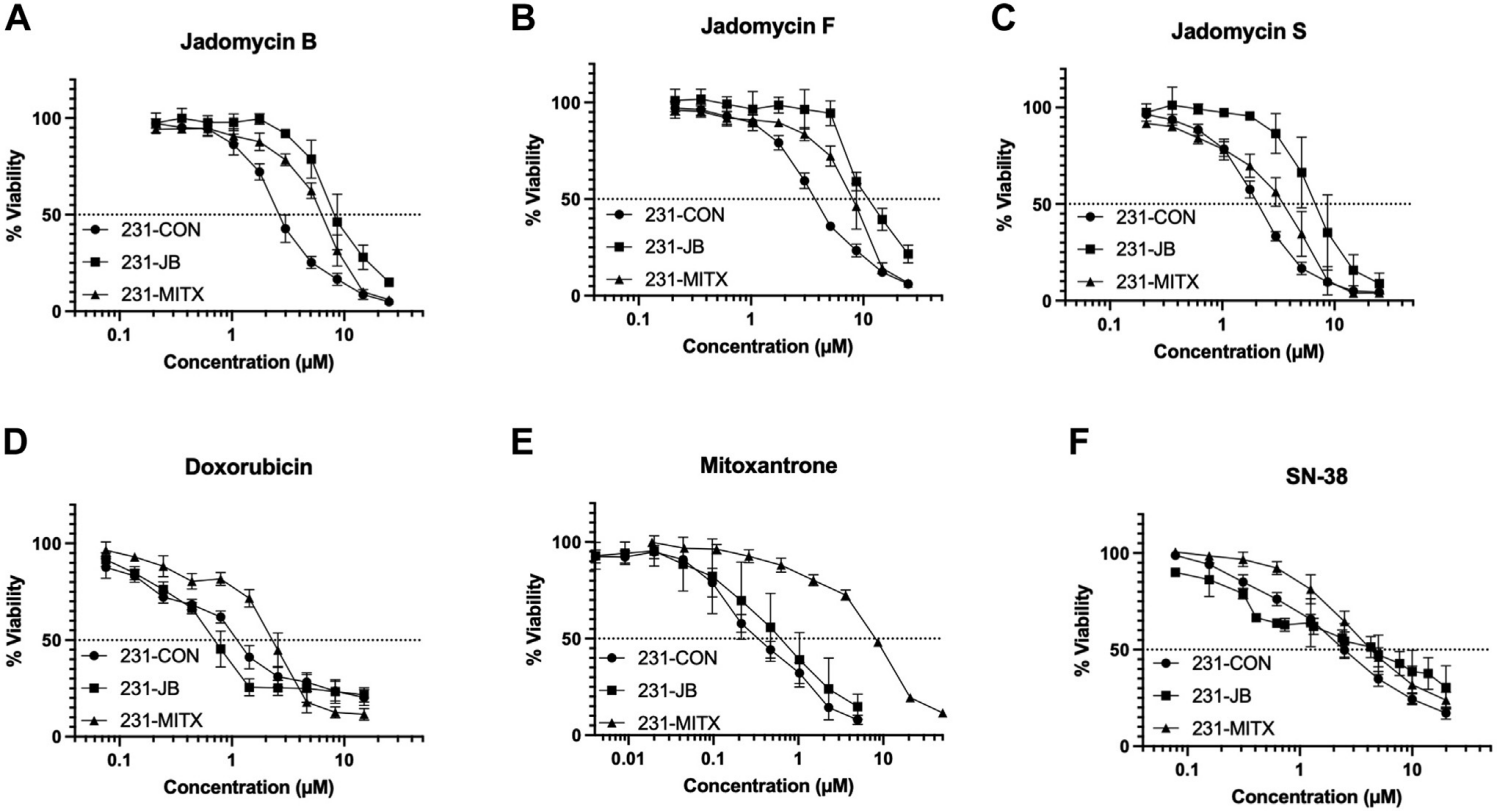
Dose-response curves for jadomycin and control drug cytotoxicity in resistant cells. Dose-response curves for 231-CON, 231-JB and 231-MITX cells exposed to (A) jadomycin B (0.21–25 *μ*M), (B) jadomycin F (0.21–25 *μ*M), (C) jadomycin S (0.21–25 *μ*M), (D) doxorubicin (0.07–15 *μ*M), (E) mitoxantrone (0.004–50 *μ*M), and (F) SN-38 (0.07–20 *μ*M) for 48 hours. Data points represent mean value of quadruplicate assays, each consisting of quadruplicate technical replicates, and expressed as % viability of unexposed controls.

Jadomycin B–resistant 231-JB cells and mitoxantrone-resistant 231-MITX cells were successfully established from 231-CON cells. Following the development of resistance, dose-response curves shifted to the right in 231-JB cells in response to jadomycins B, F, and S (Fig. 2, A–C). A significant right shift in the dose-response curves was not observed in 231-JB cells treated with doxorubicin, mitoxantrone, or SN-38 (Fig. 2, D–F).

The calculated IC_50_ values from the MTT assays are presented in Table 1. The 231-JB cells developed a low degree of resistance to jadomycin B, with a 3.2-fold higher concentration needed to elicit a 50% reduction in cellular viability compared with 231-CON cells. Furthermore, 231-JB cells demonstrated a similar degree of resistance to jadomycins S (3.39-fold) and F (3.15-fold), while the potency of mitoxantrone (1.81-fold), doxorubicin (0.71-fold), and SN-38 (1.52-fold) was not significantly changed. These comparator drugs were chosen because they target the previously suggested jadomycin B targets TOP2 (mitoxantrone and doxorubicin) and topoisomerase-1 (TOP1) (SN-38) and because mitoxantrone is primarily an ABCG2 (and possibly an ABCB1 and ABCC1) transport protein substrate (Martinez-Farina et al, 2015; Hall et al, 2017).

**Table 1:** The cytotoxic effects of control drugs and jadomycins in drug sensitive and drug-resistant cells IC_50_ values are reported with 95% CIs as calculated from dose-response curves for 231-CON, 231-JB, and 231-MITX cells (Fig. 1). Each IC_50_ value was determined from quadruplicate assays, each consisting of quadruplicate technical replicates.

Cytotoxic Drug	231-CON			231-JB			231-MITX		
	IC_50_ (95% CI)	SD	Fold Resistance	IC_50_ (95% CI)	SD	Fold Resistance	IC_50_ (95% CI) *μ*M	SD	Fold Resistance
	*μM*			*μM*			*μM*		
Doxorubicin	1.22 (0.73 to 1.72)	0.31	NA	0.87 (0.51 to 1.23)	0.23	0.71	2.14 (1.59 to 2.69)∗	0.34	1.75
Jadomycin B	2.83 (2.34 to 3.32)	0.31	NA	9.03 (5.82 to 12.24)∗	2.02	3.19	5.89 (4.94 to 6.85)∗	0.60	2.08
Jadomycin F	3.81 (3.63 to 4.00)	0.12	NA	12.01 (9.68 to 14.34)∗	1.46	3.15	7.49 (5.67 to 9.31)∗	1.14	1.96
Jadomycin S	2.08 (1.87 to 2.29)	0.13	NA	7.05 (2.84 to 11.27)∗	2.65	3.39	3.01 (1.71 to 4.31)	0.82	1.45
Mitoxantrone	0.42 (0.19 to 0.64)	0.14	NA	0.75 (−0.30 to 1.81)	0.66	1.81	6.55 (4.75 to 8.35)∗	1.45	15.75
SN-38	2.82 (2.14 to 3.51)	0.55	NA	4.30 (0.65 to 7.95)	2.29	1.52	5.15 (4.10 to 6.21)	0.42	1.83

NA, not applicable.

∗ Significant difference (*P* ≤ .05) was determined by one-way ANOVA followed by Bonferroni multiple comparison test.

In contrast to 231-JB cells, concentration-response curves for mitoxantrone-resistant 231-MITX cells shifted to the right in response to mitoxantrone (Fig. 2E). This represented a 15.75-fold increase in the IC_50_ of mitoxantrone, while potency of jadomycins B (2.08-fold), S (1.45-fold), and F (1.96-fold) was reduced to a lesser extent.

### 3.2 Association of resistance and increased mRNA expression of ABC transporters

**Fig. 3:**
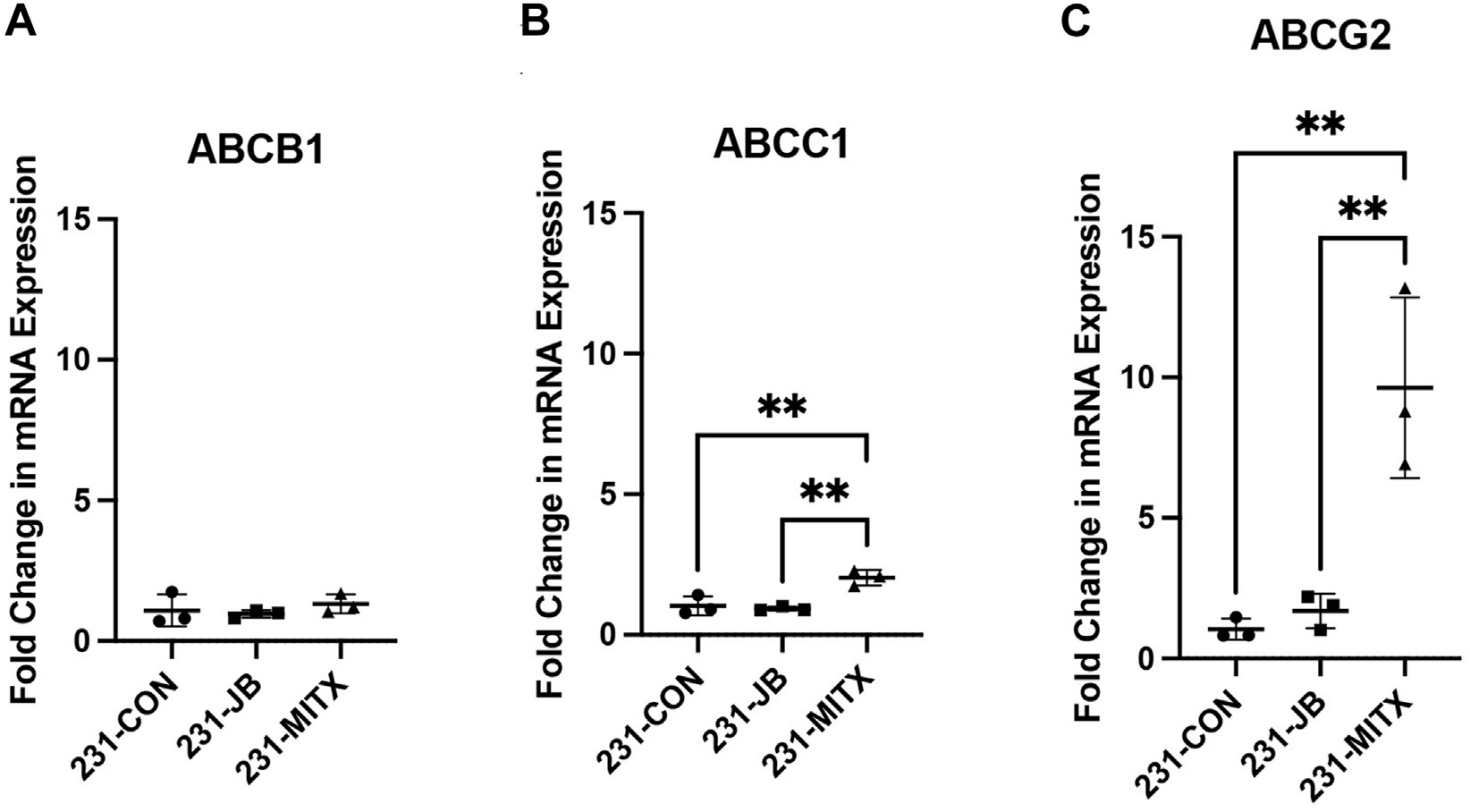
Resistance to mitoxantrone, but not jadomycin B, is associated with increased *ABCG2* mRNA. Reverse transcription quantitative PCR was used to measure baseline mRNA expression of (A) *ABCB1*, (B) *ABCC1*, and (C) *ABCG2* in 231-CON, 231-JB, and 231-MITX cells. Data points represent the mean value ± SD of triplicate assays, each consisting of duplicate technical replicates and expressed as fold change in mRNA expression compared with that of 231-CON cells. Significant difference (∗∗*P* ≤ .01) was determined by one-way ANOVA, followed by Bonferroni multiple comparison test.

No significant difference in mRNA expression was detected between 231-JB and 231-CON cells for *ABCB1*, *ABCC1*, nor *ABCG2* (Fig. 3). In 231-MITX cells, *ABCG2* expression increased 9.6-fold compared with that in 231-CON cells. RNA expression of *ABCB1* was not significantly changed in 231-MITX cells, and expression of *ABCC1* in 231-MITX cells increased 2.0-fold compared with 231-CON or 231-JB cells.

### 3.3 Changes in TOP2 mRNA expression in 231-JB and 231-MITX cells

**Fig. 4:**
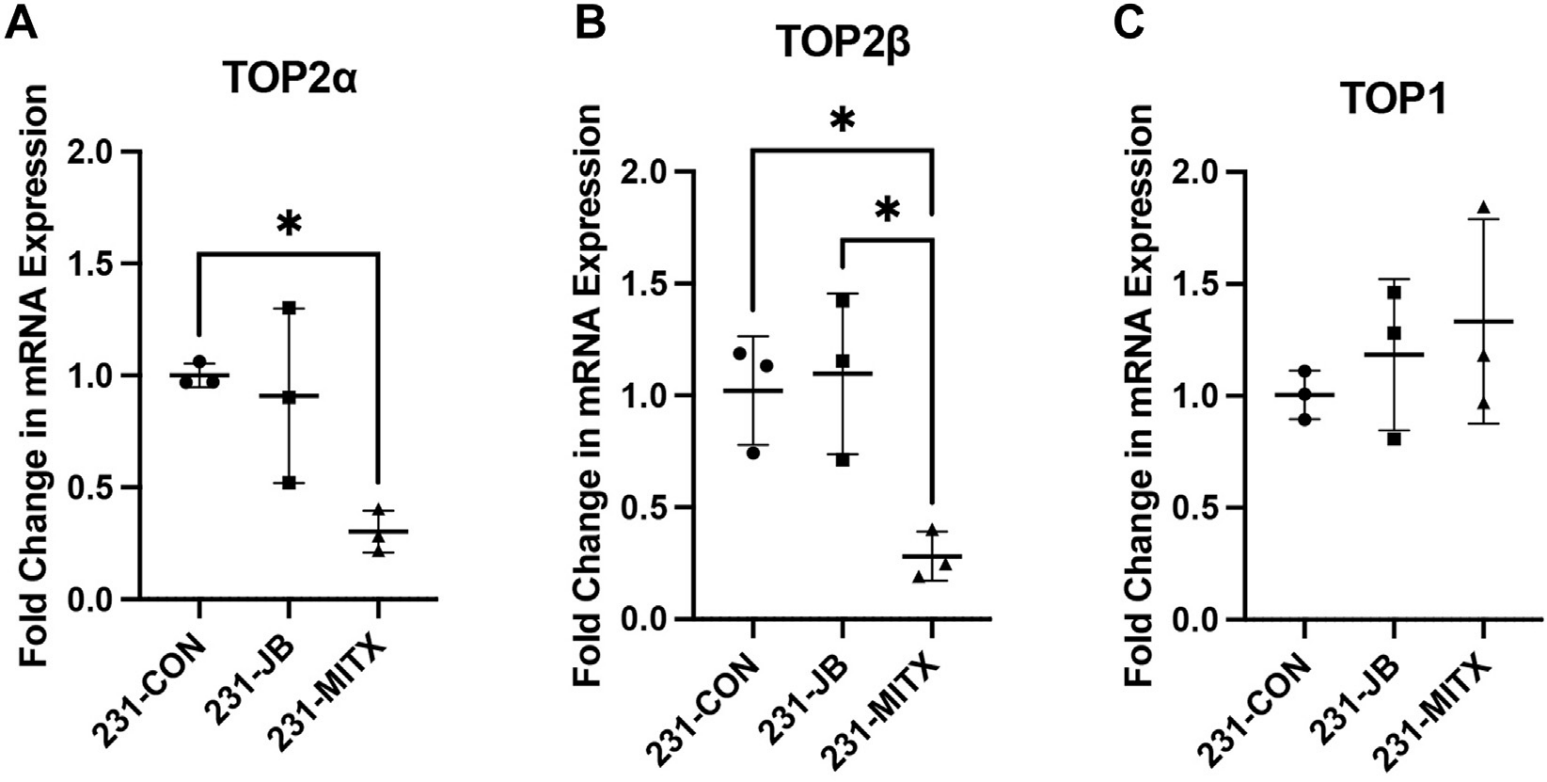
*TOP2* mRNA expression does not significantly change in 231-JB cells. Reverse transcription quantitative PCR was used to measure baseline mRNA expression of (A) *TOP2α*, (B) *TOP2β*, and (C) *TOP1* in 231-CON, 231-JB, and 231-MITX cells. Data points represent the mean value ± SD of triplicate assays, each consisting of duplicate technical replicates and expressed as fold change in mRNA expression compared with that of 231-CON cells. Significant difference (∗*P* ≤ .05) was determined by one-way ANOVA, followed by Bonferroni multiple comparison test.

With jadomycin-resistant 231-JB cells developed, topoisomerase mRNA expression was assayed to determine whether changes to *TOP1* or *TOP2* are associated with decreased jadomycin B potency. If jadomycin B primarily acts as a TOP2 poison as previously proposed, then decreased *TOP2* expression in resistant cells would be expected (Martinez-Farina et al, 2015; Hall et al, 2017). Conversely, should *TOP2* expression remain unchanged in 231-JB cells, this would add further evidence to suggest that jadomycin B does not primarily exert its cytotoxicity through interaction with TOP2 (McKeown and Goralski, 2025). No significant difference was observed in mRNA expression of either *TOP2α* or *TOP2β* in 231-JB cells compared with that in 231-CON (Fig. 4, A and B). Conversely, in 231-MITX cells *TOP2α* and *TOP2β* mRNA expression was reduced to 30% and 28%, respectively, compared with 231-CON cells. No significant difference in mRNA expression of *TOP1* was observed between 231-CON and 231-JB or 231-MITX cells (Fig. 4C).

### 3.4 Changes in mRNA expression of human cancer drug targets in 231-JB cells

**Fig. 5:**
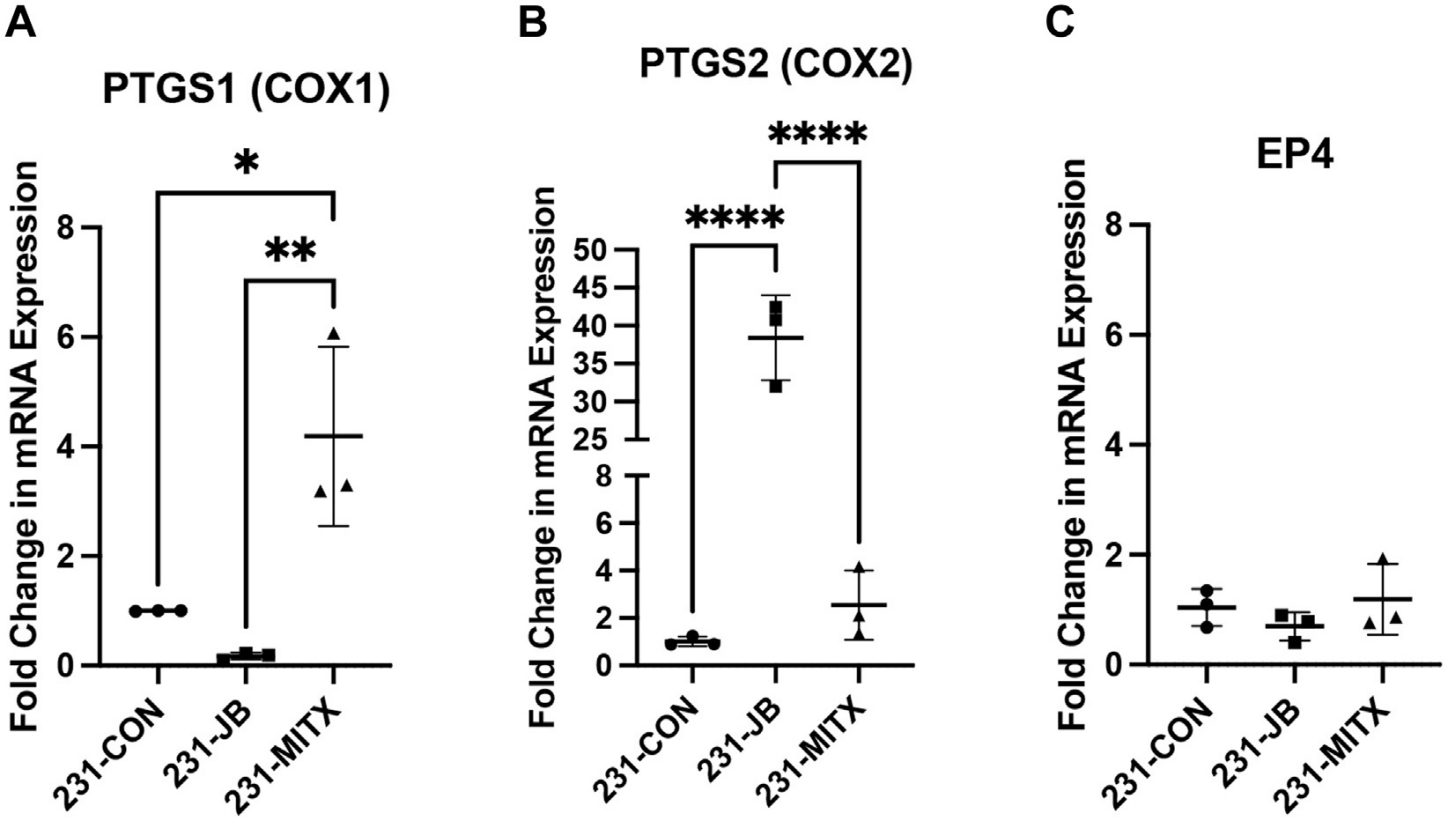
Expression of *PTGS2* mRNA is increased in 231-JB cells. Reverse transcription quantitative PCR was used to measure baseline mRNA expression of (A) *PTGS1*, (B) *PTGS2*, and (C) *EP4* in 231-CON, 231-JB, and 231-MITX cells. Data points represent the mean value ± SD of triplicate assays, each consisting of duplicate technical replicates and expressed as fold change in mRNA expression compared with that of 231-CON cells. Significant difference (∗*P* ≤ .05; ∗∗*P* ≤ .01; ∗∗∗∗*P* < .0001) was determined by one-way ANOVA, followed by Bonferroni multiple comparison test.

An 84-gene Human Cancer Drug Targets RT^2^ Profiler PCR Array was used to compare gene expression in 231-CON and 231-JB cells to identify genes contributing to jadomycin B resistance. Of the 84 genes assayed the greatest change, approximately 22-fold, was found to occur in *PTGS2*, which encodes for cyclooxygenase-2 (COX-2; Table 2). To confirm the change in *PTGS2*, mRNA was quantified by quantitative PCR in 3 independent experiments (Fig. 5). *PTGS2* exhibited a 38.4-fold increase in expression in 231-JB cells compared with that in 231-CON cells, while no significant change was observed in 231-MITX cells. *PTGS1* expression, coding for cyclooxygenase-1 (COX-1), was not significantly changed in 231-JB cells compared with that in 231-CON cells, but did significantly increase to 4.2-fold of control in 231-MITX cells. There was no significant change in prostaglandin E2 receptor (*EP4*) mRNA expression in drug-resistant cells.

**Table 2:** Expression changes found in human cancer drug targets screen Changes in gene expression found with the QIAGEN 84 gene Human Cancer Drug Targets PCR array. Fold change in 231-JB cells was normalized to expression in 231-CON cells. Results reported are from a single comparison, and therefore, no statistical analysis was conducted.

Gene	Description	Fold Change
***PTGS2***	**Prostaglandin-endoperoxide synthase 2**	**22.33**
*CTSS*	Cathepsin S	3.95
*CTSL1*	Cathepsin L1	3.59
*ESR2*	Estrogen receptor 2	3.51
*FLT4*	Fms-related tyrosine kinase 4	3.33
*ERBB4*	V-erb-a erythroblastic leukemia viral oncogene homolog 4	2.94
*KIT*	V-kit Hardy-Zuckerman 4 feline sarcoma viral oncogene homolog	2.38
*CTSD*	Cathepsin D	2.25
*IGF1*	Insulin-like growth factor 1	2.22
*RHOB*	Ras homolog gene family, member B	2.20
***TXNRD1***	**Thioredoxin reductase 1**	**2.12**
*HDAC4*	Histone deacetylase 4	1.95
***AURKC***	**Aurora kinase C**	**1.95**
*PRKCD*	Protein kinase C, delta	1.93
*NTN3*	Netrin 3	1.90
*FIGF*	C-fos induced growth factor	1.76
*PLK3*	Polo-like kinase 3	1.57
*CDC25A*	Cell division cycle 25 homolog A	1.55
*PGR*	Progesterone receptor	1.48
*TXN*	Thioredoxin	1.46
*GAPDH*	Glyceraldehyde-3-phosphate dehydrogenase	1.44
*IRF5*	Interferon regulatory factor 5	1.42
*TP53*	Tumor protein p53	1.36
*ATF2*	Activating transcrition factor 2	1.38
*FLT1*	Fms-related tyrosine kinase 1	1.36
*TNKS*	Tankyrase, TFR1-interacting ankyrin-related ADP-ribose polymerase	1.34
*HDAC6*	Histone deacetylase 6	1.32
*MDM4*	Mdm4 p53 binding protein homolog	1.29
*CDK7*	Cyclin-dependent kinase 7	1.27
*HIF1A*	Hypoxia inducible factor 1, *α* subunit	1.26
*MDM2*	Mdm2 p53 binding protein homolog	1.21
*EGFR*	Epidermal growth factor receptor	1.17
*CTSB*	Cathepsin B	1.15
*B2M*	*β*2-Microglobulin	1.15
*HDAC1*	Histone deacetylase 1	1.13
*AKT1*	V-akt murine thymoma viral oncogene homolog 1	1.09
*CDK4*	Cyclin-dependent kinase 4	1.08
*HRAS*	V-Ha-ras Harvey rat sarcoma viral oncogene homolog	1.08
*PRKCA*	Protein kinase C, *α*	1.07
*CDK9*	Cyclin-dependent kinase 9	1.06
*AKT2*	V-akt murine thymoma viral oncogene homolog 2	1.05
*IGF1R*	Insulin-like growth factor 1 receptor	1.04
*NFKB1*	Nuclear factor kappa light polypeptide gene enhancer in B-cells 1	1.03
*PARP4*	Poly (ADP-ribose) polymerase family, member 4	1.03
*PARP1*	Poly (ADP-ribose) polymerase 1	1.02
***ABCC1***	**ATP-binding cassette, sub-family C, member 1**	**1.01**
*CDK5*	Cyclin-dependent kinase 5	1.01
*MTOR*	Mechanistic target of rapamycin	1.01
***TOP2B***	**Topoisomerase II *β* 180kDa**	**1.00**
*NRAS*	Neuroblastoma RAS viral oncogene homolog	1.00

**Gene**	**Description**	**Fold Change**

*TERT*	Telomerase reverse transcriptase	0.33
*PDGFRB*	Platelet-derived growth factor receptor, *β* polypeptide	0.45
*CDK1*	Cyclin-dependent kinase 1	0.46
*IGF2*	Insulin-like growth factor 2	0.56
*BIRC5*	Baculoviral IAP repeat contianing 5	0.60
*HDAC11*	Histone deacetylase 11	0.60
*PRKCB*	Protein kinase C, *β*	0.62
*KDR*	Kinase insert domain receptor	0.63
*PDGFRA*	Platelet-derived growth factor receptor, *α* polypeptide	0.66
*ERBB3*	V-erb-b2 erythroblastic leukemia viral oncogene homolog 3	0.68
*PIK3CA*	Phosphoinositide-3-kinase, catalytic, *α* polypeptide	0.72
*CDK8*	Cyclin-dependent kinase 8	0.73
*KRAS*	V-Ki-ras2 Kirsten rat sarcoma viral oncogene homolog	0.76
*RPLP0*	Ribosomal protein, large, P0	0.77
*ESR1*	Estrogen receptor 1	0.78
*HDAC8*	Histone deacetylase 8	0.78
*PLK4*	Polo-like kinase 4	0.79
*CDK2*	Cyclin-dependent kinase 2	0.80
*HDAC3*	Histone deacetylase 3	0.82
*HDAC2*	Histone deacetylase 2	0.83
*RHOA*	Ras homolog gene family, member A	0.84
*ACTB*	Actin, *β*	0.84
*HDAC7*	Histone deacetylase 7	0.85
*PLK2*	Polo-like kinase 2	0.85
***AURKB***	**Aurora kinase B**	**0.87**
*PRKCE*	Protein kinase C, epsilon	0.88
*BCL2*	B-cell CLL/lymphoma 2	0.88
*PLK1*	Polo-like kinase 1	0.89
*HSP90AA1*	Heat shock protein 90kDa *α*, class A member 1	0.91
***TOP2A***	**Topoisomerase II *α* 170kDa**	**0.91**
*HPRT1*	Hypoxanthine phosphoribosyltransferase 1	0.93
*PIK3C2A*	Phosphoinositide-3-kinase, class 2, *α* polypeptide	0.94
*GRB2*	Growht factor receptor-bound protein 2	0.95
*PIK3C3*	Phosphoinositide-3-kinase, class 3	0.95
***AURKA***	**Aurora kinase A**	**0.96**
*GSTP1*	Glutathione S-transferase *π*1	0.96
*ERBB2*	V-erb-b2 erythroblastic leukemia viral oncogene homolog 2	0.96
*HSP90B1*	Heat shock protein 90kDa *β*, member 1	0.97
*PARP2*	Poly(ADP-ribose) polymerase 2	0.98

### 3.5 COX-2 protein expression in 231-JB cells

**Fig. 6:**
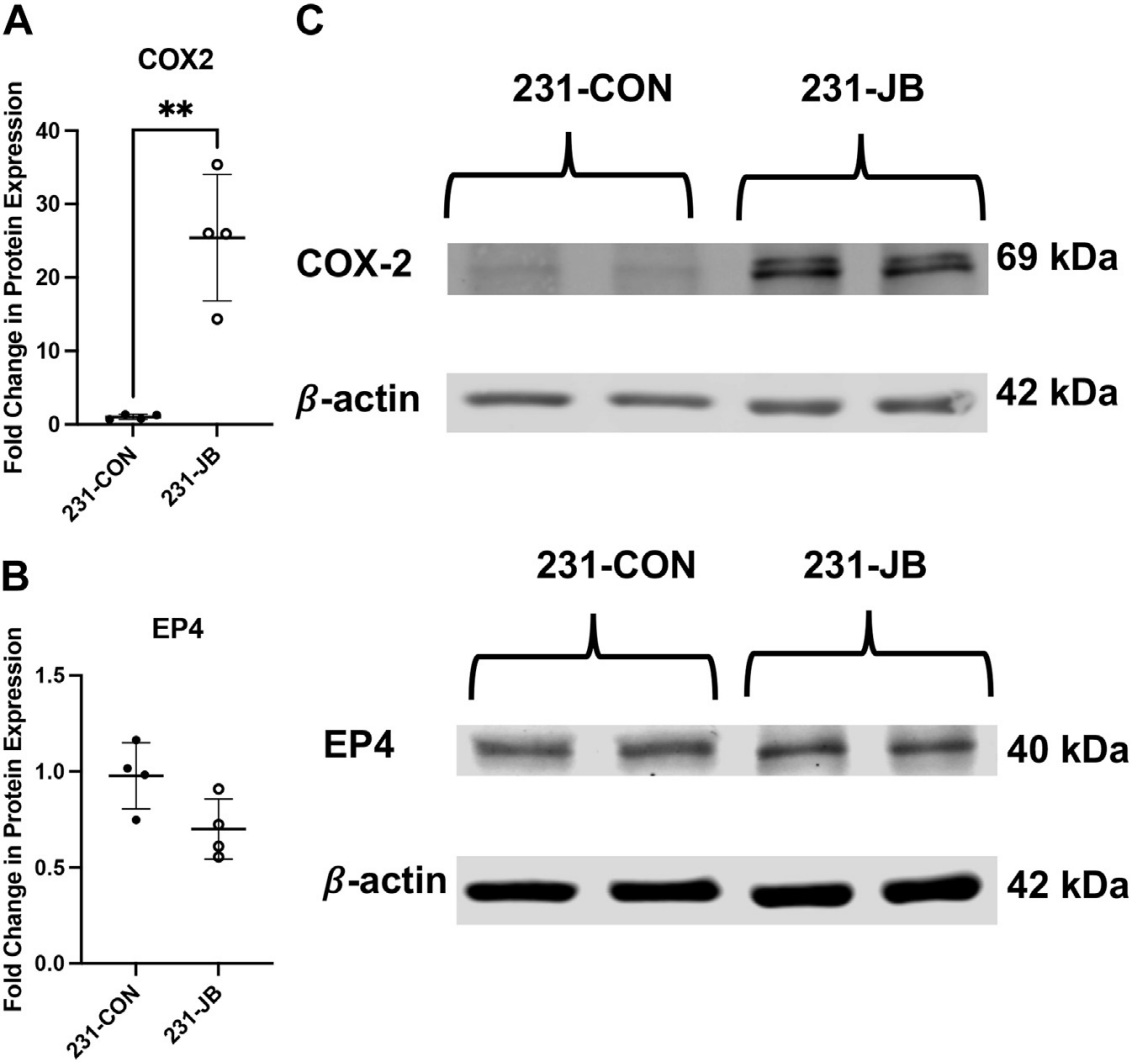
Expression of COX-2 protein increased in 231-JB cells. Protein expression of baseline (A) COX-2 and (B) EP4 in 231-CON and 231-JB cells was measured by Western blot. Representative blots (C) are shown. Data points represent the mean values ± SD of quadruplicate assays, each consisting of duplicate technical replicates and expressed as fold change in protein expression compared with that of 231-CON cells. Significant difference (∗∗*P* ≤ .01) was determined by unpaired *t* test.

Increases in RNA expression are not always correlated with a corresponding increase in protein expression (Maier et al, 2009). Protein expression of COX-2 and EP4 were therefore quantified in 231-CON and 231-JB cells. COX-2 protein was increased by 25.4-fold in 231-JB cells compared with that in control, while no significant change was observed in EP4 protein expression (Fig. 6).

### 3.6 Selective and nonselective COX-2 inhibitors reverse jadomycin B resistance

**Fig. 7:**
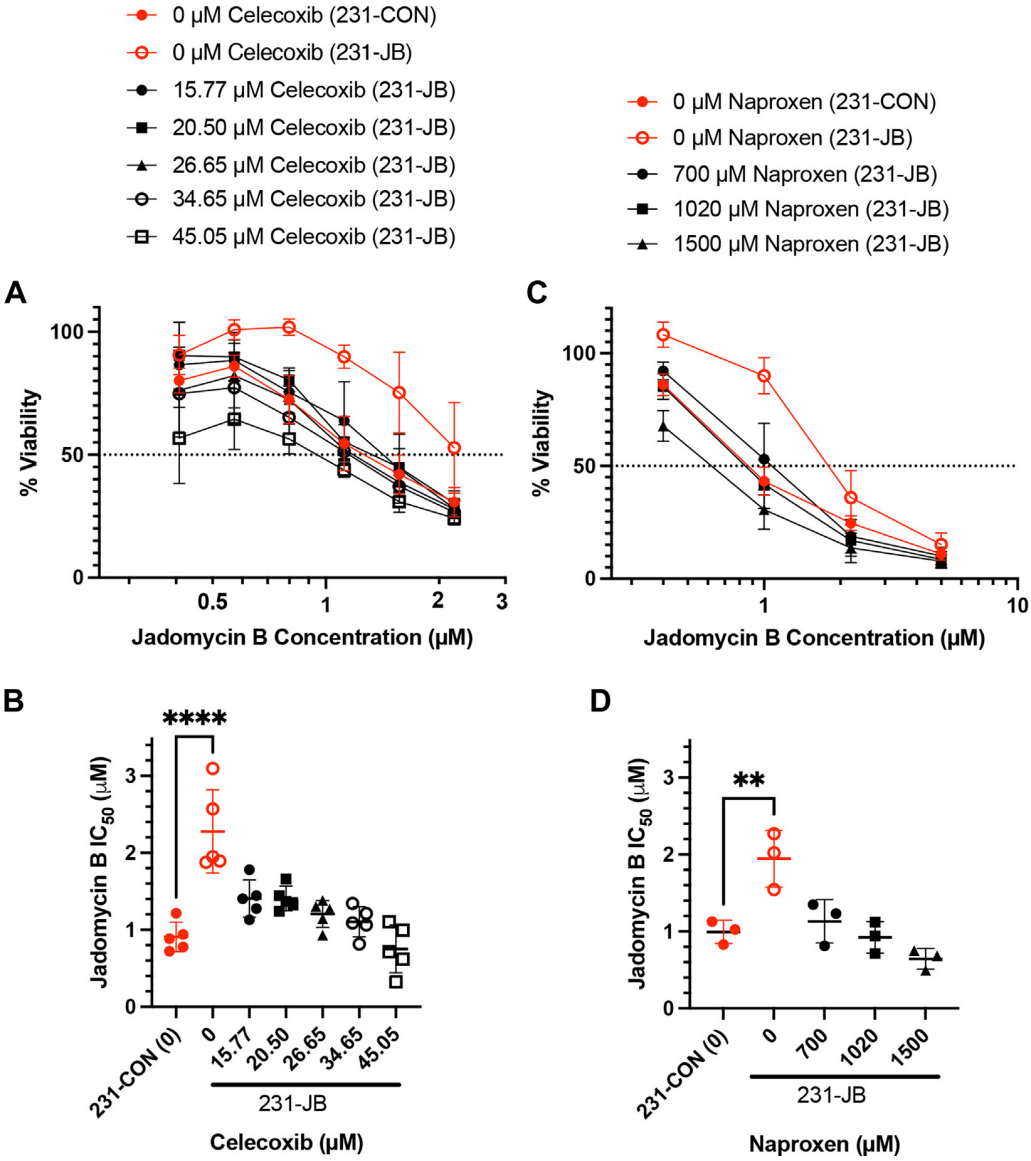
Pharmacological inhibition of COX-2 reverses 231-JB resistance to jadomycin B. IC_50_ values of 231-JB cells exposed to the COX-2 inhibitors celecoxib (0–45 *μ*M) or naproxen (0–1500 *μ*M) as measured by MTT assay are shown as mean values ± SD of quintuplicate assays, each consisting of quadruplicate technical replicates and expressed as concentration of jadomycin B in micromoles per liter compared with that of 231-CON cells (red, closed circles). Concentration-response curves (A, C) used to generate IC_50_ values (B, D) are also shown. Significant difference (∗∗*P* ≤ .01; ∗∗∗∗*P* < .0001) was determined by one-way ANOVA, followed by Bonferroni multiple comparison test.

If increased expression of COX-2 is responsible for the observed increase in resistance to jadomycin B in 231-JB cells, then pharmacological inhibition of COX-2 should reverse the resistance. In the absence of any COX-2 inhibitor, 231-JB cells demonstrated a right shift in the jadomycin B concentration cell viability response curve (Fig. 7, A and C) and corresponding increase in IC_50_ compared with 231-CON cells (Fig. 7, B and D). Adding the COX-2 selective inhibitor celecoxib resulted in a concentration-dependent left shift in the jadomycin B concentration response curve in the 231-JB cells (Fig. 7A) and a reduction in IC_50_s to levels that were similar to the 231-CON cells (Fig. 7B). Similar results were observed with the nonselective COX-1/COX-2 inhibitor naproxen (Fig. 7, C and D).

### 3.7 Celecoxib sensitizes 231-CON and MCF-7 cells to jadomycin B

**Fig. 8:**
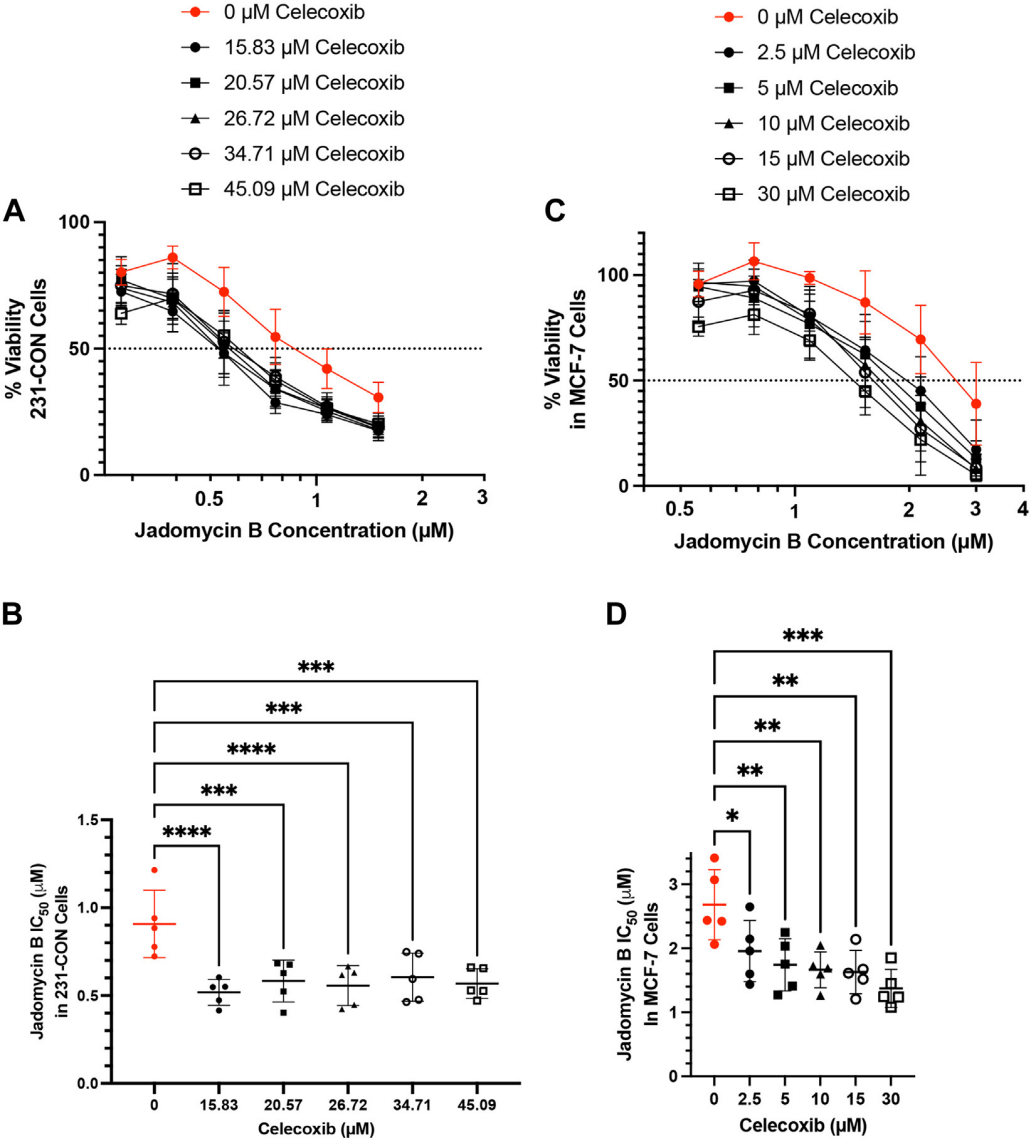
Celecoxib sensitizes 231-CON and MCF-7 cells to jadomycin B. IC_50_ values of 231-CON and MCF-7 cells exposed to a combination of jadomycin B (0–1.5 *μ*M or 0.3 *μ*M, respectively) and celecoxib (0–45 *μ*M or 0–30 *μ*M, respectively) as measured by MTT assay are shown as mean values ± SD of quintuplicate assays, each consisting of quadruplicate technical replicates and expressed as concentration of jadomycin B in micromoles per liter compared with those of the combination with celecoxib vehicle control (red, closed circles). Concentration-response curves for 231-CON cells (A) and MCF-7 cells (C) used to generate IC_50_ values for 231-CON cells (B) and MCF-7 cells (D) are also shown. Significant difference (∗*P* ≤ .05; ∗∗*P* ≤ .01; ∗∗∗*P* ≤ .001; ∗∗∗∗*P* ≤ .0001) was determined by one-way ANOVA, followed by Bonferroni multiple comparison test.

Given the reduction in resistance observed in the combination of jadomycin B with pharmacological inhibitors of COX-2 in the 231-JB cells, it was pertinent to determine whether celecoxib could further sensitize 231-CON cells to the cytotoxic effect of jadomycin B. 231-CON cells were exposed to combinations of jadomycin B and celecoxib (Fig. 8, A and B). As with the resistant 231-JB cells, addition of celecoxib resulted in a left shift in the jadomycin B cytotoxicity–response curve, leading to a reduction in IC_50_ values. To determine whether this effect extends to other breast cancer cell subtypes known to have inducible COX-2, hormone receptor–positive MCF-7 cells were also assayed (Liu and Rose, 1996). MCF-7 cells showed a similar left shift in the jadomycin B cytotoxicity–response curve and a significant reduction in IC_50_ values in the presence of celecoxib (Fig. 8, C and D).

### 3.8 Jadomycin B decreases accumulation of PGE_2_ in 231-CON cells

**Fig. 9:**
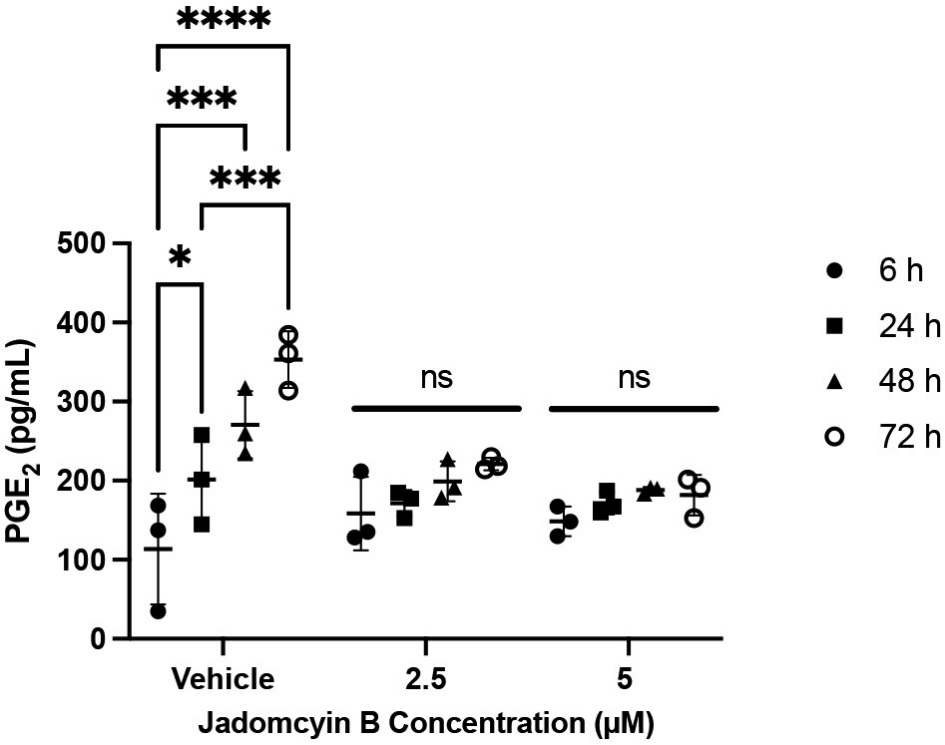
Jadomycin B prevents accumulation of PGE_2_ in cell growth medium. Concentration of PGE_2_ measured in growth medium of 231-CON cells exposed to vehicle control or 2.5 or 5 *μ*M jadomycin B for 6, 24, 48, or 72 hours as measured by ELISA are shown as mean values ± SD of triplicate assays, each consisting of duplicate technical replicates and expressed as concentration of PGE_2_ in picograms per milliliter. Significant difference (∗*P* ≤ .05; ∗∗∗*P* ≤ .001; ∗∗∗∗*P* ≤ .0001) from 6-hour time point was determined by one-way ANOVA, followed by Bonferroni multiple comparison test. ns, not significant.

As increases in the RNA and protein expression of COX-2 were observed to be associated with jadomycin resistance, and this resistance could be reversed with COX-2 inhibitors, the next logical step was to assay the level of PGE_2_ produced by 231-CON cells in the presence of jadomycin B. PGE_2_ is a major downstream product of arachidonic acid metabolism by COX-2. If jadomycin B inhibits COX-2 activity, a reduction in PGE_2_ levels would be expected. In cells exposed to vehicle control PGE_2_ levels in the growth medium increased to 177%, 239%, and 312% of the 6-hour baseline after 24, 48, and 72 hours, respectively (Fig. 9). Following exposure to either 2.5 or 5.0 *μ*M jadomycin B, there was no significant increase from the 6-hour baseline levels of PGE_2_ at any measured time point.

## 4 Discussion

In a recent study, we showed that jadomycin B has different pharmacological effects on the cell cycle and different pharmacological interactions with TOP2 (mitoxantrone and doxorubicin) and TOP1 (SN-38) poisons and the protease inhibitor MG132 than known TOP2 poisons (McKeown and Goralski, 2025). Specifically, jadomycin B arrested cells in S phase, whereas mitoxantrone arrested cells in G_2_/M phase. Furthermore, jadomycin B acted additively/synergistically in combination with the protease inhibitor MG132, while doxorubicin and mitoxantrone were antagonistic with MG132 (McKeown and Goralski, 2025). These results further indicated that TOP2 poisoning is not the primary mechanism of jadomycin action. To determine what other processes may be involved in the cytotoxic effect of jadomycin B, the establishment of a jadomycin-resistant human triple-negative breast cancer cell line became the specific goal of this work. To that end, the development and characterization of 231-JB is herein described.

A low degree, approximately 3-fold, of jadomycin resistance was developed in 231-JB cells. Cell lines established from postchemotherapy patients typically have a 2–5-fold increase in resistance, suggesting that a 3-fold increase is within the clinically relevant range (McDermott et al, 2014). Jadomycin B, F, and S demonstrated a similar level of resistance, suggesting that resistance to jadomycins is mediated through a shared feature within the unchanged jadomycin backbone as opposed to the amino acid substituent group. This unique observation demonstrates for the first time that resistance to 1 jadomycin conveys resistance to others. However, it is not known whether the pattern of cross-resistance occurs with more diverse jadomycin structures (MacLeod et al, 2018). Additionally, while 231-JB cells showed cross-resistance to other jadomycins, there was no cross-resistance to TOP2 (mitoxantrone and doxorubicin), nor TOP1 (SN-38) poisons. In comparison, the 231-MITX cells exhibited a 15.8-fold increase in resistance to mitoxantrone, with only a 2-fold increase in resistance to jadomycins B or F and not S. It is well established that resistance to mitoxantrone can be mediated by increased ABCG2 expression (McDermott et al, 2014). 231-JB cells showed no significant change in *ABCB1*, *ABCC1*, or *ABCG2*, while 231-MITX cells were shown to overexpress *ABCG2* as expected. This observation corroborates that jadomycin B is a poor ABCG2 substrate and resistance is not expected to occur with ABCG2 overexpression.

Having established that differences in resistance are exhibited by 231-JB and 231-MITX cells, it was important to characterize the possible ways in which that resistance arose. If jadomycin B cytotoxicity were dependent on an interaction with TOP2, it would be reasonable to assume that 231-JB cells would also have decreased TOP2 expression, preventing enzyme poisoning. Short-duration treatment with high concentrations of jadomycins B, F, and S (20 *μ*M for 36 hours) and mitoxantrone (1 *μ*M for 36 hours) have previously been shown to decrease expression of *TOP2α* and *TOP2β* mRNA in 231-CON cells, so it was important to determine if a similar change occurs in conjunction with chronic, low-dose jadomycin B exposure, leading to jadomycin resistance (Hall et al, 2017). While 231-MITX cells exhibited the expected decrease in *TOP2α* and *TOP2β* mRNA expression, with no effect on *TOP1*, 231-JB cells showed no significant change in *TOP2α* nor *TOP2β*. High concentration, short-duration exposure to jadomycin B may be eliciting a nonspecific effect on *TOP2*, resulting from toxicity to the cells, which is absent in the low dose, chronic exposure used to select for resistance in the present work. As with the previously discussed data on cell cycle and synergy showing differing effects on the cell cycle and a differing profile of interaction with the protease inhibitor MG132, this lack of effect on topoisomerase gene expression following the establishment of jadomycin resistance provides greater evidence that jadomycin B is acting on some other target (or targets) at low concentrations known to induce cytotoxicity (McKeown and Goralski, 2025).

Using a drug-resistant cancer cell line is an established approach for determination of the mechanism through which cytotoxicity is affected (McDermott et al, 2014). A human cancer drug target array was used to screen for changes in gene expression in 231-JB cells compared with that in 231-CON cells. This screen matched previously discussed results, showing no change to *TOP2α*, *TOP2β*, or *ABCC1* mRNA expression in 231-JB cells. Importantly, the largest change identified was an increase in expression of *PTGS2*, which codes for the COX-2 protein. The increase in expression of both *PTGS2* mRNA and COX-2 protein were therefore verified in 231-JB cells. The final experiment to associate the involvement of COX-2 with 231-JB cell resistance to jadomycin B was the pharmacological inhibition of COX-2. All concentrations of celecoxib and naproxen tested resulted in a decrease in the concentration of jadomycin B needed to induce 50% cell death in 231-JB cells, with similar results observed in nonresistant 231-CON and MCF-7 cells exposed to either celecoxib or naproxen, further supporting an interaction between COX-2 function and jadomycin cytotoxicity. Furthermore, jadomycin B treatment prevented the accumulation of PGE_2_ in the cell culture medium, suggesting a functional inhibition of COX-2 activity.

COX-1 and COX-2 are the rate-limiting enzymes in the conversion of arachidonic acid to various prostaglandins (Chandrasekharan and Simmons, 2004; Simmons et al, 2004; Majumder et al, 2018; Ching et al, 2020). Of specific importance is PGE_2_ as this is the most abundant prostaglandin in cancer (Ching et al, 2020). The PGE_2_ pathway has been identified as being of particular importance in breast cancer due to its tumor-promoting effects and role in the development of a cancer stem cell phenotype (Kundu et al, 2014; Walker et al, 2021). Once produced and released, PGE_2_ can bind to any member of a group of G-protein–coupled receptors responsible for the activation of intracellular signaling cascades; these are known as PGE_2_ receptors 1 through 4 (EP1–4) (Sugimoto and Narumiya, 2007; Reader et al, 2011). EP4 is commonly upregulated in many cancers including those of the breast (Majumder et al, 2018; Ching et al, 2020). When activated, EP4 initiates intracellular signaling to promote cell survival, proliferation, and the acquisition of cancer stem cell-like characteristics (Kundu et al, 2014; Majumder et al, 2015; Xu et al, 2018; Ching et al, 2020). EP4 activation has also been associated with increased breast cancer cell invasion and metastasis through epidermal growth factor receptor transactivation, which can be blocked by EP4 antagonists in murine models of breast cancer (Ma et al, 2006; Tönisen et al, 2017).

The increased expression of COX-2, decreased accumulation of PGE_2_, and unchanged expression of EP4 described in this work suggest that 231-JB cell survival is mediated through increased prostaglandin signaling via COX-2 production. While our results support the importance of COX-2, a role for COX-1 cannot yet be ruled out, and thus, further research is needed on the specific involvement of COX-1. Of specific interest is that the 231-JB cell line overexpresses COX-2 without a correlated increase in ABCB1, ABCC1, or ABCG2 transporter expression. ABCB1, ABCC1, and ABCG2 transporter overexpression and COX-2 overexpression are typically linked in cancer (Surowiak et al, 2008; Szczuraszek et al, 2009; Zeliha et al, 2020). The 231-JB cells represent a possible model for investigating the molecular mechanisms underlying that linkage because of their unusual disconnection between COX-2 and ABC transporter expression. Determining the way(s) in which 231-JB cells differ from other multidrug-resistant cells, which have simultaneous upregulation of COX-2 and ABC transporters may offer insight into the different ways in which multidrug-resistance can develop. For this purpose, 231-JB cells could be used to study the role of COX-2 in multidrug resistance in a role completely apart from functional studies of jadomycin B activity.

## 5 Conclusion

Jadomycins are natural products, which continue to demonstrate promising anticancer activity. This work has shown for the first time that 231-JB can be selected for, leading to a unique phenotype distinct from that gained with resistance to mitoxantrone. The 231-JB cells exhibit a unique resistance where *ABCG2* expression is not upregulated with increased COX-2 expression, and they therefore may be useful in exploring how and why COX-2 and ABCG2 expression are typically connected in chemotherapy resistance. By establishing a jadomycin-resistant human breast cancer cell line, COX-2 was identified as a potential target through which jadomycin B exerts its anticancer effect. The continued examination of the interaction between jadomycin B and the COX-2 enzyme is an important future direction, which will help further characterize the effect of jadomycin B in human breast cancer cells.

## Financial support

This work was supported by operating grants from the Dalhousie Pharmacy Endowment (B.T.M. and K.B.G.) and the 10.13039/100007669Beatrice Hunter Cancer Research Institute (K.B.G.) and the 10.13039/501100000038Natural Sciences and Engineering Research Council of Canada (D.L.J.). B.T.M. was a trainee in the Cancer Research Training Program of the Beatrice Hunter Cancer Research Institute, with funds provided by the Saunders Matthey Foundation for Cancer Prevention, Terry Fox Research Institute, and the Natural Sciences and Engineering Research Council of Canada [CREATE Grant 510963].

## Conflict of interest

The authors declare no conflicts of interest.
