# Supplementary material for: Acquired resistance to jadomycin B in human triple-negative breast cancer cells is associated with increased cyclooxygenase-2 expression
Source: J Pharmacol Exp Ther. 2025 Mar 27;392(5):103565. doi: 10.1016/j.jpet.2025.103565 (PMC12163490; doi:10.1016/j.jpet.2025.103565)
Supplement: Supplementary Tables 1-2 [file mmc1.docx]

**Title:** Acquired Resistance to Jadomycin B in Human Triple Negative Breast Cancer Cells is Associated with Increased Cyclooxygenase-2 Expression

Supplemental Material

**Authors:** Brendan T. McKeown, Brandon Groves, David L. Jakeman, and Kerry B. Goralski

**Journal:** The Journal of Pharmacology and Experimental Therapeutics

**Manuscript Number:** JPET-D-24-00016R1

Supplemental Table 1: PCR primers used to determine expression of relevant genes in 231-CON, 231-JB, and 231-MITX cells

Supplemental Table 2: Primary and secondary antibodies used for immunoblotting assays
